# Growth and Survival of Bagged *Lucilia sericata* Maggots in Wounds of Patients Undergoing Maggot Debridement Therapy

**DOI:** 10.1155/2013/192149

**Published:** 2013-07-25

**Authors:** Helena Čičková, Marek Čambal, Milan Kozánek, Peter Takáč

**Affiliations:** ^1^Institute of Zoology, Slovak Academy of Sciences, Dúbravská cesta 9, 845 06 Bratislava, Slovakia; ^2^Scientica s. r. o., Hybešova 33, 831 06 Bratislava, Slovakia; ^3^1st Department of Surgery, University Hospital, Mickiewiczova 13, 813 69 Bratislava, Slovakia; ^4^Faculty of Medicine, Comenius University, Špitálska 24, 813 72 Bratislava, Slovakia

## Abstract

Maggot debridement therapy (MDT) is an established method of debridement of nonhealing wounds. Despite intense clinical research about its efficacy and effects of substances produced by the larvae, growth and development of maggots in the wounds remain largely unexplored. In the present study, the bags with larvae (*n* = 52), which had been used to debride traumatic, ischemic, diabetic and venous ulcers, were collected and examined. Survival, length, width and larval instar of the maggots within each bag were recorded and analyzed with respect to the wound type and duration of the treatment. Survival of maggots after a 48-h cycle of MDT ranged between 63.6 and 82.7%. Maggots in venous ulcers had on average 9–19% higher mortality than maggots within traumatic, ischemic, and diabetic ulcers. Length of larvae after 48 h cycle of MDT reached on average 7.09–9.68 mm, and average width varied between 1.77 and 2.26 mm. Larvae in venous ulcers were significantly smaller after 48 h, but not after 72 h treatment compared to the other wound types. Further studies should be aimed to identify other patient-associated factors which might influence growth and survival of the larvae during maggot debridement therapy.

## 1. Introduction

Maggot debridement therapy is an accepted method of biosurgical debridement. Many clinical trials have shown that maggot debridement therapy, also referred to as “biosurgery” or “larval therapy,” results in faster wound debridement when compared to conventional treatments [1–3]. Its beneficial effects were reported especially in the management of suppurative or intractable wounds, when conventional methods of wound treatment had failed or were contraindicated [4–6].

Recent years have brought about renewed interest in maggot therapy. Maggots were shown to produce an array of antimicrobial and tissue growth-promoting factors, enzymes, and other biologically active substances [7–10], which aid in wound healing.

Despite intense research in this area [9], most studies have concentrated on the underlying mechanisms responsible for improved debridement and healing, such as production of antibacterial substances, growth factors, and digestive enzymes. Little interest has been given to describing maggot development in the wound.

The life cycle of the blow fly *Lucilia sericata*, whose larvae are typically used in MDT, starts with the egg stage, followed by three larval instars, a “wandering” larval stage, pupa, and eventually adult fly. The fact that larvae consume only necrotic tissue while leaving healthy structures intact has been exploited in larval therapy which is, in fact, a controlled therapeutic myiasis [11].

While development of the maggots has been thoroughly explored under laboratory conditions [12–14], there are still major gaps in the understanding of their development under restricting conditions of MDT. The present study examines some aspects of larval development in biobags during larval therapy in patients with different types of wounds.

## 2. Materials and Methods

The majority of the patients who participated in this study were indicated for minor or major amputation of the limb, and maggot debridement therapy (MDT) was offered only if previous attempts to heal the wounds with standard treatment modalities (hydrogel, hydrocolloid dressings, enzymatic debridement, antibacterials) had failed. No selection criteria were applied; each patient indicated for MDT who agreed with the treatment could participate (clean wounds and ulcers with massive *Pseudomonas aeruginosa *infection were not indicated for MDT). Most of the wounds were diabetic foot ulcers, while a minority of patients had traumatic, ischemic, and venous wounds. Patients were treated in the 1st Department of Surgery, University Hospital, and Faculty of Medicine, Comenius University in Bratislava, Slovakia, in both ambulatory and hospital settings. The experimental protocol was approved by the Ethical committee of the University Hospital and Faculty of Medicine, Comenius University (Reference number 112/2005). All patients gave informed consent before the beginning of MDT. The study was performed in accordance with the Declaration of Helsinki (2004). 

Although we initially intended to compare larval development under both conditions of larval therapy (i.e., free-range and bagged maggots), when presented with a choice, the patients typically requested contained technique with larvae sealed in a nylon bag. This method was also preferred by the medical and nursing staff due to convenient application and removal of maggots from the wound as well as positive experience with its efficacy in wound debridement. During the course of this study (18 months), only one patient with diabetic ulcer was treated using the free-range technique.

Bags with sterile larvae (*L. sericata*) were obtained from MEDALT (Bernolákova 1/A, Malacky, Slovakia), a nonprofit organization, and were provided free of charge to the patients participating in the study. Bags containing young second-instar larvae were prepared individually for each patient to match the size of the wound based on the physician's instructions. Dimensions of the bags ranged from 4 × 4 cm to 13.5 × 9.5 cm. The total number of maggots in the bags differed according to the size of the bags, but always corresponded to 5 larvae/cm^2^ of the folded bag surface area. This was the standard dosage of bagged larvae supplied by the manufacturer (MEDALT, personal communication); other dosages were available upon specific requests. Bagged larvae were supplied in sterile plastic containers and placed in a transportation box (AcuTemp; 23 cm wide × 20.5 cm long × 25 cm high) together with 6 cooling pads (ClimSel C 7N; Climator, Sweden), previously cooled to 5-6°C.

The bags were applied for approximately 48 or 72 hours (range: 46–49 and 69–72 hours, resp.), depending on the patient's tolerance and medical personnel's evaluation of the wound. Brief information regarding the patients (age and sex), wounds (duration, size, depth, location, etiology, and treatment prior to application of larvae), dimensions of the bag, and length of the MDT cycle were recorded (Table 1). The condition of the wound was carefully monitored during the treatment. Each wound received one bag with larvae during the MDT cycle, except for a patient with complicated traumatic injury of the hand and wrist where due to the size and irregular shape of the wound 3 bags had to be applied.

Following the treatment, each bag was removed from the wound, placed in a sterile plastic box, and frozen at −20°C for 2-3 days to kill the larvae. Dead maggots disintegrate very quickly under the aggressive conditions within wounds; the larvae which had died prior to being placed in a freezer could be easily distinguished from survivors by dark color of the larval fat body and/or low turgor and were not included in the analyses. Each frozen bag was then opened in aseptic conditions; maggots were removed, submersed in ≈0.5% sodium hypochlorite for 2-3 minutes, and washed with distilled water. Maximum length and width of each maggot were measured to the nearest 0.1 mm with a micrometer, and larval instar of each maggot from the bag was determined based on the morphology of the anterior and posterior spiracles [15] under a dissecting microscope. Total number of maggots in the bag was used to calculate survival of the larvae for a given wound type and duration of treatment. A total of 52 bags were analyzed (Table 1).

### 2.1. Statistical Analysis

Data on larval survival and width were power-transformed (*X*′ = *X* ^2^) to meet the criteria of statistical analyses. The data (larval survival, lengths, and widths) in venous and diabetic wounds were analyzed with 2-way analysis of variance (type III sum of squares) with wound type and treatment duration as experimental factors, while parameters in all 4 types of wounds after 48 hr treatment were evaluated with one-way analysis of variance. Tukey-Kramer procedure was used to separate means which were significantly different [16].

Statistical analyses were carried out in *R*, version 2.15.2 [17].

## 3. Results and Discussion

After 48 hours of larval therapy, average survival of larvae in the bags in the different types of wounds ranged between 63.6 and 82.7% (Table 2). Survival in traumatic and venous wounds was extremely variable, ranging between 33.7 and 91.0% in traumatic wounds and 25.0 and 86.3% in venous ulcers. Overall, no significant differences in larval survival between the four wound types were observed after 48 hours (*F* = 1.274, df = 3, 18, *P* = 0.3130). On the other hand, significant differences in length (*F* = 7.875, df = 3, 18, *P* = 0.0015) and width (*F* = 3.707, df = 3,18, *P* = 0.0309) of the larvae were observed after 48 hours of larval therapy. Larvae from venous ulcers were shorter (7.09 mm) and thinner (1.77 mm) than in traumatic, ischemic, or diabetic ulcers (8.43–9.68 mm long and 2.11–2.26 mm wide; Table 3). Proportion of immature second-instar larvae after 48 h treatment was also markedly higher in venous wounds (Table 2); however, a preliminary heterogeneity *χ* ^2^-testing showed significant differences in proportions of second instars between replicates (i.e., patients) which hindered pooling and further analysis of the data.

A closer examination of larval development in venous and diabetic wounds after 48 and 72 h treatment showed that survival in venous ulcers was on average 19% lower than in diabetic wounds, but the etiology of the wound just failed to reach statistical significance at the 95% level (wound type: *F* = 3.7243, df = 1, 37, *P* = 0.0613; duration of treatment: *F* = 1.5662, df = 1, 37, *P* = 0.2186; interaction: *F* = 0.040, df = 1, 37; *P* = 0.8427). However, significant differences were observed in length (wound type: *F* = 17.6021, df = 1, 37, *P* = 0.0002, duration of treatment; *F* = 0.5233, df = 1,37, *P* = 0.4740, interaction: *F* = 11.4613, df = 1,37, *P* = 0.0017; Table 3) and width of larvae (wound type: *F* = 5.1055, df = 1, 37, *P* = 0.0298, length of treatment: *F* = 2.3467, df = 1, 37, *P* = 0.1341, interaction: *F* = 1.5062, df = 1, 37, *P* = 0.2275; Table 3). While larvae in venous ulcers were smaller and with a higher proportion of second instar larvae than in diabetic ulcers after 48 h MDT, the difference in size after 72 hours was no longer significant.

Information regarding survival and development of the surgical maggots in wounds of patients undergoing larval therapy has been very scarce, but it may be an important guide for medical practitioners when assessing the number of maggots necessary to successfully debride the wound, as well as duration of the treatment.

Wolff and Hansson mention that “the larvae seemed to thrive especially well in the wounds of diabetic patients, which were all completely debrided” [5]. Indeed, we observed high survival and growth rates of maggots in diabetic foot ulcers. Similar results were also observed in the wounds of traumatic and ischemic origin. It was very interesting to see that the larvae in venous ulcers did not grow so quickly and their survival was much lower than in the other types of wounds. Despite these differences, however, most of the venous ulcers were visually well debrided and one cycle of MDT was usually sufficient to remove necrotic tissue.

Several factors could be responsible for the observed differences in larval growth and survival: (1) differences in the amount and quality of necrotic tissue between the different types of wounds. Larvae of *L. sericata*, which are typically used for MDT and were also used in the present study, feed only on dead tissue and once it has been consumed, the larvae starve. While we did not estimate the amount of necrotic tissue in the wounds, some authors point out that venous ulcers in particular often contain little slough [1], so the slow growth and high mortality might be the result of larval starvation. It may also be possible that the necrotic tissue present in venous ulcers has lower nutritional value for the larvae. (2) Bacterial load within the wounds. In the house fly (*Musca domestica*), which has also been used in larval therapy [18], certain bacteria are associated with reduced growth and high mortality rates, while others promote larval development [19, 20]. In the blow fly *L. sericata*, which is typically used for wound debridement, available data about effects of bacteria on larval growth and survival are limited to a single bioassay with *Staphylococcus aureus* which did not find any effect of the bacterium on larval development [21]. However, it has been reported that certain bacteria affect immune responses of the maggots [22], so the potential effect of microorganisms on larval survival and development should not be neglected. (3) Different metabolic conditions within the wound bed and presence of substances that inhibit larval growth or are toxic to the larvae. The underlying disorder may result in conditions which are not suitable for larval development [23] and/or might affect the nutritional quality of necrotic tissue. It may also be possible that medications taken by some of the patients might have influenced the results. Residues of some wound dressings were shown to negatively affect larval survival and growth [24]. On the other hand, Sherman et al. [25] found that the most commonly used antibiotics do not influence survival and growth of larvae at levels typically reached in human blood during therapy. Most of the patients in this study were, however, elderly people, and many of them were treated with multiple drugs during MDT. Patients with venous ulcers, where we observed decreased survival and reduced growth, were treated for chronic venous insufficiency and other associated cardiovascular diseases, dyslipidemia, and coxarthrosis, but these diseases were also present in the patients in other treatment groups (except for the patients with traumatic injuries which were considered otherwise healthy). Detailed analysis of patient-associated factors and drugs taken by the patients may reveal possible associations between larval development and growth but was beyond the scope of the present investigation due to the limited number of patients enrolled in this study.

Size of the maggots upon removal of the bags from wounds, which is reported in this study, is lower than typical dimensions of fully grown larvae. Grassberger and Reiter [12] report that larvae of *L. sericata* may reach 15-16 mm at 34°C and with ample food source. While in some of the bags from diabetic and ischemic ulcers we recorded maximum larval length of 12-13 mm after a 48 h MDT cycle, the majority of the larvae within the bag were usually smaller. A small number of “undersized” second-instar larvae were present in most of the bags. As shown by Thomas et al. [14], it is very likely that the larvae did not reach their full size because of the restrictive conditions of MDT. The most important limiting factor determining the size of maggots is the total amount of dead tissue present in the wound. Secondly, being placed in a bag, the larvae are contained in a relatively small area and cannot move to places outside the bag, which may still contain slough. Moreover, the bag itself limits the amount of tissue that can be consumed by the maggots. Mean weight of maggots (and thus their size) as the result of containment in a bag may be reduced to 13 mg compared to the 20 mg in their free-range counterparts after 48-hour incubation, and their survival may be reduced by over 10% [14]. As the larvae grow, their nutritional requirements increase and the passage of liquefied necrotic tissue through the bag wall may become insufficient to sustain their further development.

It has been generally recommended and also stated in the manufacturers' guidelines to keep the larvae in the wound for 3-4 days [1, 13]. However, our results show that, while the bagged larvae in venous ulcers still increase in size after 48 hours of MDT application, the same trend is not observed in diabetic wounds. In fact, the 72 h old larvae from diabetic ulcers were slightly shorter and thicker compared to the 48 h old larvae. This may indicate that the maggots reached their final size and entered the postfeeding “wandering” stage when the larvae primarily search for suitable dry pupation sites. The wandering stage may, at 34°C, appear as early as 48 hours after hatching of the larvae from the eggs [12]. Thus, for diabetic ulcers, it may not be reasonable to continue the MDT cycle beyond 48–72 hours.

This study does not advocate the use of contained technique over the free-range method. In fact, traditional larval therapy with loose larvae results in faster debridement and required less applications and less total maggots to full wound debridement than the contained technique [1, 26].

While we realize that sample size in the current study was relatively small which may limit generalization of the results and that our study would be much more powerful if it also included information about development of free-range larvae, our data indicate that there may be significant differences in the growth and development of larvae in different types of wounds. 

## 4. Conclusions

Our results indicate the presence of marked differences in development of bagged larvae of *Lucilia sericata* in different types of wounds. Further studies should be aimed to identify other patient-associated factors which might influence growth and survival of the larvae during maggot debridement therapy.

## Figures and Tables

**Table 1:** Patients and wound characteristics.

	Wound type					
	Traumatic	Ischemic	Venous		Diabetic	
Duration of MDT cycle	48 hours	48 hours	48 hours	72 hours	48 hours	72 hours
Total number of analyzed bags	4	7	4	9	7	21
*Patient characteristics *						
Age						
<60 years (%)	4 (100.0)	1 (14.3)	1 (25.0)	2 (22.2)	4 (57.1)	0 (0.0)
>60 years (%)	0 (0.0)	6 (85.7)	3 (75.0)	7 (77.8)	3 (42.9)	21 (100.0)
Sex						
Male (%)	4 (100.0)	7 (100.0)	1 (25.0)	2 (22.2)	7 (100.0)	4 (19.0)
Female (%)	0 (0.0)	0 (0.0)	3 (75.0)	7 (77.8)	0 (0.0)	17 (81.0)
*Wound characteristics *						
Size (%)						
<15 cm^2^ (%)	0 (0.0)	1 (14.3)	2 (50.0)	3 (33.3)	0 (0.0)	0 (0.0)
15–50 cm^2^ (%)	1 (25.0)	3 (42.9)	2 (50.0)	2 (22.2)	5 (71.4)	17 (81.0)
>50 cm^2^ (%)	3^4^ (75.0)	3 (42.9)	0 (0.0)	4 (44.4)	2 (28.6)	4 (19.0)
Location						
Hand and wrist (%)	3^4^ (75.0)	0 (0.0)	0 (0.0)	0 (0.0)	0 (0.0)	0 (0.0)
Foot (%)	0 (0.0)	1 (14.3)	0 (0.0)	0 (0.0)	7 (100.0)	20 (95.2)
Ankle (%)	1 (25.0)	0 (0.0)	3 (75.0)	3 (33.3)	0 (0.0)	1 (4.8)
Calf (%)	0 (0.0)	6 (85.7)	1 (25.0)	6 (66.7)	0 (0.0)	0 (0.0)
Duration						
<3 months (%)	4 (100.0)	1 (14.3)	4 (100.0)	4 (44.4)	5 (71.4)	13 (61.9)
>3 months (%)	0 (0.0)	6 (85.7)	0 (0.0)	5 (55.6)	2 (28.6)	8 (38.1)
Depth^1^						
Superficial (%)	1 (25.0)	0 (0.0)	4 (100.0)	9 (100.0)	0 (0.0)	2 (9.5)
Deep (%)	3^4^ (75.0)	7 (100.0)	0 (0.0)	0 (0.0)	7 (100.0)	19 (90.5)
Treatment preceding^2^ MDT						
Systemic and local antibiotic/antimycotic therapy, surgical debridement (%)	3^4^ (75.0)	6 (85.7)	0 (0.0)	0 (0.0)	4 (57.1)	9 (42.9)
Local antiseptic treatment only (%)	0 (0.0)	1 (14.3)	3 (75.0)	2 (22.2)	1 (14.3)	7 (33.3)
Local antiseptic treatment and natural products^3^ (%)	0 (0.0)	0 (0.0)	1 (25.0)	6 (66.7)	2 (28.6)	4 (19.0)
Enzymatic debridement (%)	0 (0.0)	0 (0.0)	0 (0.0)	0 (0.0)	0 (0.0)	1 (4.8)
Autolytic debridement (%)	1 (25.0)	0 (0.0)	0 (0.0)	1 (11.1)	0 (0.0)	0 (0.0)

^1^Wounds were classified as superficial if they affected only epidermal and dermal layers and deep if they included tendons or bones.

^2^Up to 7 days prior to application of bagged maggots.

^3^Natural products applied topically included medicinal honey and herbal extracts.

^4^A single wound over 180 cm^2^ of irregular shape was treated with 3 bags (>50 cm^2^ each) placed on different parts of the wound.

**Table 2:** Survival and development of *L. sericata* larvae in wounds of different etiology.

Wound type	Mean (95% CI^1^) survival (%) of maggots following MDT cycle of		Mean (range) proportion (%) of second instars among all larvae following MDT cycle of	
	48 hours	72 hours	48 hours	72 hours
Traumatic	72.8 (0.0–100.0)	—	0.6 (0.0–1.1)	—
Ischemic	79.1 (68.3–88.6)	—	0.8 (0.0–2.4)	—
Venous	63.6 (0.0–92.1)	55.9 (27.0–74.4)	7.3 (1.4–20.0)	0.7 (0.0–3.2)
Diabetic	82.7 (69.4–94.2)	74.7 (68.9–80.1)	0.7 (0–1.7)	0.6 (0.0–2.5)

^1^Confidence interval.

**Table 3:** Growth of *L. sericata* larvae developing in wounds of different etiology.

Wound type	Mean (95% CI^1^) length (mm) of larvae following MDT cycle of		Mean (95% CI) width (mm) of larvae following MDT cycle of	
	48 hours	72 hours	48 hours	72 hours
Traumatic	8.43^ab2^ (6.89–9.97)	—	2.11^cd^ (1.66–2.49)	—
Ischemic	9.68^a^ (8.92–10.44)	—	2.12^cd^ (1.87–2.38)	—
Venous	7.09^bA^ (5.79–8.40)	9.08^aB^ (8.33–9.83)	1.77^cC^ (0.99–2.30)	2.31^cC^ (2.18–2.42)
Diabetic	9.47^aA^ (8.49–10.45)	9.19^aA^ (8.81–9.57)	2.26^dC^ (2.13–2.37)	2.45^cC^ (2.29–2.61)

^1^Confidence interval.

^2^means within columns marked with the same lowercase letters and means within rows marked with the same uppercase letters are not significantly different at *P* = 0.05.

## References

[1] Dumville JC, Worthy G, Bland JM (2009). Larval therapy for leg ulcers (VenUS II): randomised controlled trial. *British Medical Journal*.

[2] Opletalová K, Blaizot X, Mourgeon B (2012). Maggot therapy for wound debridement: a randomized multicenter trial. *Archives of Dermatology*.

[3] Zarchi K, Jemec GB (2012). The efficacy of maggot debridement therapy—a review of comparative clinical trials. *International Wound Journal*.

[4] Mumcuoglu KY, Ingber A, Gilead L (1999). Maggot therapy for the treatment of intractable wounds. *International Journal of Dermatology*.

[5] Wolff H, Hansson C (2003). Larval therapy—an effective method of ulcer debridement. *Clinical and Experimental Dermatology*.

[6] Orkiszewski M, Madej J, Kilian T The use of maggot therapy as an adjunct to surgical debridement: a paedriatic case report. http://www.worldwidewounds.com/2006/march/Orkiszewski/Maggot-Therapy-Paediatric-Surgery.html.

[7] van der Plas MJA, Baldry M, van Dissel JT, Jukema GN, Nibbering PH (2009). Maggot secretions suppress pro-inflammatory responses of human monocytes through elevation of cyclic AMP. *Diabetologia*.

[8] Bexfield A, Bond AE, Morgan C (2010). Amino acid derivatives from *Lucilia sericata* excretions/secretions may contribute to the beneficial effects of maggot therapy via increased angiogenesis. *British Journal of Dermatology*.

[9] Nigam Y, Dudley E, Bexfield A, Bond AE, Evans J, James J (2010). The physiology of wound healing by the medicinal maggot, *Lucilia sericata*. *Advances in Insect Physiology*.

[10] Telford G, Brown AP, Kind A, English JSC, Pritchard DI (2011). Maggot chymotrypsin I from *Lucilia sericata* is resistant to endogenous wound protease inhibitors. *British Journal of Dermatology*.

[11] Sherman RA (2009). Maggot therapy takes us back to the future of wound care: new and improved maggot therapy for the 21st century. *Journal of Diabetes Science and Technology*.

[12] Grassberger M, Reiter C (2001). Effect of temperature on *Lucilia sericata* (Diptera: Calliphoridae) development with special reference to the isomegalen- and isomorphen-diagram. *Forensic Science International*.

[13] Blake FAS, Abromeit N, Bubenheim M, Li L, Schmelzle R (2007). The biosurgical wound debridement: experimental investigation of efficiency and practicability. *Wound Repair and Regeneration*.

[14] Thomas S, Wynn K, Fowler T, Jones M (2002). The effect of containment on the properties of sterile maggots. *British Journal of Nursing*.

[15] Shewell GE, McAlpine JF (1987). Calliphoridae. *Manual of Nearctic Diptera*.

[16] Zar JH (2010). *Biostatistical Analysis*.

[17] R Core Team (2012). *R: A Language and Environment For Statistical Computing*.

[18] Li Q, Lu R, Huo R, Fu H (2009). Maggots of *Musca domestica* in treatment of acute intractable wound. *Surgery*.

[19] Schmidtmann ET, Martin PA (1992). Relationship between selected bacteria and the growth of immature house flies, *Musca domestica*, in an axenic test system. *Journal of Medical Entomology*.

[20] Zurek L, Schal C, Watson DW (2000). Diversity and contribution of the intestinal bacterial community to the development of *Musca domestica* (Diptera: Muscidae) larvae. *Journal of Medical Entomology*.

[21] Barnes KM, Gennard DE (2011). The effect of bacterially-dense environments on the development and immune defences of the blowfly *Lucilia sericata*. *Physiological Entomology*.

[22] Kawabata T, Mitsui H, Yokota K, Ishino K, Oguma K, Sano S (2010). Induction of antibacterial activity in larvae of the blowfly *Lucilia sericata* by an infected environment. *Medical and Veterinary Entomology*.

[23] Renner R, Treudler R, Simon JC (2008). Maggots do not survive in pyoderma gangrenosum. *Dermatology*.

[24] Thomas S, Andrews A (1999). The effect of hydrogel dressings on maggot development. *Journal of Wound Care*.

[25] Sherman RA, Wyle FA, Thrupp L (1995). Effects of seven antibiotics on the growth and development of *Phaenicia sericata* (Diptera: Calliphoridae) larvae. *Journal of Medical Entomology*.

[26] Steenvoorde P, Jacobi CE, Oskam J (2005). Maggot debridement therapy: free-range or contained? An in-vivo study. *Advances in Skin & Wound Care*.

